# Effects of Delaying the Storage of ‘Hass’ Avocados under a Controlled Atmosphere on Skin Color, Bioactive Compounds and Antioxidant Capacity

**DOI:** 10.3390/plants13111455

**Published:** 2024-05-24

**Authors:** Daniela Olivares, Pablo A. Ulloa, Cristina Vergara, Ignacia Hernández, Miguel Ángel García-Rojas, Reinaldo Campos-Vargas, Romina Pedreschi, Bruno G. Defilippi

**Affiliations:** 1 Instituto de Investigaciones Agropecuarias, INIA-La Platina, Santa Rosa 11610, Santiago 8831314, Chile; olivaresdaniela@gmail.com (D.O.); pablo.ulloa@inia.cl (P.A.U.); cristina.vergara@inia.cl (C.V.); miguel.garcia@inia.cl (M.Á.G.-R.); 2 Facultad de Ciencias Agronómicas y de los Alimentos, Escuela de Agronomía, Pontificia Universidad Católica de Valparaíso, Calle San Francisco s/n, Quillota 2260000, Chile; ignacia.hernandez@pucv.cl (I.H.); romina.pedreschi@pucv.cl (R.P.); 3 Facultad de Ciencias Agronómicas, Centro de Estudios Postcosecha, Universidad de Chile, Santa Rosa 11315, Santiago 8820808, Chile; reinaldo.campos@uchile.cl

**Keywords:** *Persea americana*, exocarp, chlorophyll, anthocyanins, quality parameters, postharvest

## Abstract

During ripening, ‘Hass’ avocado skin changes from green to purple/black. Low-temperature storage with a controlled atmosphere (CA) is the most widely used method for avocado storage; however, few studies have simulated this technology and considered the days of regular air (RA) storage prior to CA storage. Herein, the effect of delaying the storage of ‘Hass’ avocado (>30% dry matter) in a CA was examined. Long-term storage conditions (5 °C for 50 days) corresponded to (i) regular air storage (RA), (ii) CA (4 kPa O_2_ and 6 kPa CO_2_) and (iii) 10 days in RA + 40 days in a CA and (iv) 20 days in RA + 30 days in a CA. Evaluations were performed during storage and at the ready-to-eat (RTE) stage. Skin color remained unchanged during storage, but at the RTE stage, more color development was observed for fruits stored under CA conditions, as these fruits were purple/black (>50%). At the RTE stage, the anthocyanin content increased, and compared to fruit under RA, fruit under a CA contained a five-fold greater content. A 20-day delay between harvest and CA storage increased the fruit softening rate and skin color development after cold storage, reducing the effectiveness of CA as a postharvest technology for extending storage life.

## 1. Introduction

Skin or exocarp color is an important quality attribute in avocado, and in ‘Hass’ and ‘Gem’ varieties, this factor indicates ripeness and acceptability by handlers and consumers [1,2,3,4]. Color is a basic physical property of agrofood products and can be correlated with other quality attributes, such as presence/absence of visual defects (e.g., vascular, external browning and lenticel damage) and potentially health-related characteristics [5]. Avocado tissues contain high levels of bioactive compounds such as carotenoids, vitamins B and E and phenolic compounds [6,7]. Therefore, there has been a higher interest in products derived from avocado, such as the skin and seeds, which may be an excellent source of antioxidants and other bioactive compounds [6,8]. ‘Hass’ is the most commercialized variety worldwide and is characterized by the development of skin color changes from green to dark purple during ripening [3,9,10,11]. These changes mainly result from a slight decrease in chlorophyll (green tone) content during the first few days of ripening, followed by an increase in carotenoid and anthocyanin concentrations (purple/black shades) [1,4,12,13,14]. Cyanidin-3-*O*-glucoside represents up to 95% of the total anthocyanins in ‘Hass’ avocado skin, and this pigment is responsible for the increase in anthocyanin concentration during ripening [4,15]. The pigment concentration can be influenced by agricultural management and climatic conditions. This mainly results from factors during harvest and storage, such as the maturity stage, temperature and relative humidity, time of storage or postharvest technologies [1,2,4,5]. Due to the distance between orchards and the marketplace, fruit quality must be preserved during prolonged storage periods (40–50 days) by applying postharvest technologies [1,16]. These technologies extend quality and shelf life by reducing the respiration rate and ethylene production [16,17]. Low-temperature storage in combination with a controlled atmosphere (CA) is the main technology used to extend the storage life of avocados [18]. CA storage applies decreased oxygen levels (between 2.5 and 5 kPa) and increased carbon dioxide levels (5–7.5 kPa CO_2_) in containers [19]. These low levels reduce the softening rate of the mesocarp, decrease physiological disorders and delay color development [9]. The factors that affect the efficacy of CA storage include variety, maturity stage and temperature during storage, among others [9,19]. When ‘Hass’ was stored in a CA, changes in the relative rate of softening and skin color darkening were observed after storage [19,20]. On the other hand, ‘Edranol’ and ‘Fuerte’ (avocado green skin) varieties subjected to a CA during long-term storage (50 days) showed a delay in ripening [9], while different quality parameters were maintained compared with fruits submitted to regulated air storage.

Exporting avocados to long, distant markets is a major challenge due to the overall quality requirements of the consumers [2]. Therefore, during the value chain for this species, growers and exporters must optimize crucial steps, such as cultural practices, harvest stage, temperature management and logistics between harvest and shipment. An important factor that influences the efficiency of postharvest technologies, including CA, is the time that the fruit remains at a cold temperature before the CA containers are consolidated [20]. This delay is mainly caused by incorrect postharvest handling, the long distance between the orchard and the packing facility or the low availability of CA containers, among other factors.

Thus, as countries export to distant markets, it is necessary to simulate various aspects of the ‘Hass’ avocado supply chain, including the time spent in RA storage before the CA containers are consolidated. The goal of this study was to evaluate the effect of delaying the storage of ‘Hass’ avocados in a CA on the physiological parameters, maturity and quality attributes of the fruit. The changes in skin color, a crucial quality attribute for this variety, were further studied by evaluating the changes in the concentrations of the main pigments during storage and ripening.

## 2. Results

### 2.1. Physiological and Quality Parameters

**Figure 1:**
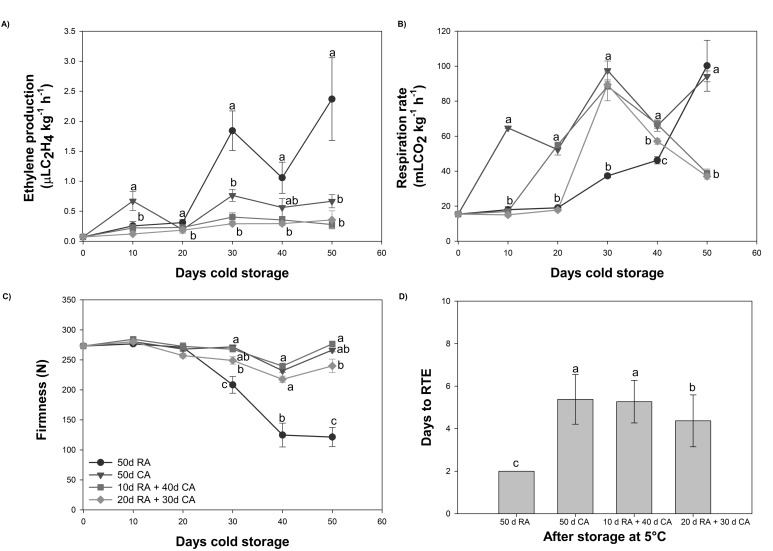
Physiological and quality parameters of avocado fruit subjected to different treatments. (**A**) Ethylene production, (**B**) respiration rate, (**C**) firmness and (**D**) days to ready-to-eat (RTE). Data represent the means of treatment ± standard errors (SE). Different letters indicate significant differences between treatments for each evaluation time (*p* < 0.05).

The physiological and maturity parameters were measured at harvest and every 10 days (10 days, 20 days, 30 days, 40 days and 50 days) during cold storage. The results are shown in Figure 1 and described below.

#### 2.1.1. Ethylene Production and Respiration Rates

Our results revealed that the level of ethylene production was low for the RA and CA treatments (Figure 1A). Throughout cold storage, slightly greater levels were observed for fruits immediately stored under a CA after harvest than fruits with a delayed CA establishment. After 30 days of storage at 5 °C, twice as much ethylene was found in the RA-stored fruit than in the fruits stored under a CA (Figure 1A). In contrast to the observations attained for ethylene production, the respiration rate was greater in fruits stored under a CA (Figure 1B). After entry into the CA, an increase in the respiration rate was observed, and the maximum level at 30 days of storage was 2.6-fold greater than that of the RA-stored fruit. Although a CA is a widely used technology for controlling respiration rates, its efficacy is affected by avocado maturity and cultivar differences, which can lead to CO_2_ and ethylene accumulation if the gas exchange is insufficient [21,22]; as a result, the rate of fruit ripening and senescence are impacted. On the other hand, a significant decrease in the CO_2_ production rate was observed after 40 and 50 days of cold storage (30.3% and 57.4%, respectively) when the CA establishment was delayed by 20 days, showing similar behavior to fruits with a 10-day delay. The CO_2_ production rate of RA-stored fruit peaked after 50 days of cold storage (Figure 1B), which was concomitant with the peak in ethylene production.

#### 2.1.2. Mesocarp Firmness

The results showed that during the first 20 days of cold storage for all treatments (CA and RA), no differences were observed in mesocarp firmness (Figure 1C). This parameter decreased in RA-stored fruit (after 30 days at 5 °C), with values reaching ~ 200 N. Firmness levels (250–270 N) close to those observed at harvest were maintained for all CA treatments (Figure 1C). Immediately after 50 days of cold storage, the firmness decreased in fruits stored with a delayed CA established for 20 days (240 N). The mesocarp firmness of the RA-stored fruit was lower than that observed at 30 days (150 N) (Figure 1C). However, a rapid decrease in firmness was observed at 20 °C for RA-treated fruits that reached the ready-to-eat stage (2 days), whereas CA-treated fruits required more time (5 days) to reach the same RTE stage (Figure 1D).

#### 2.1.3. Physiological and Pathological Disorders

In this study, no external damage (exocarp browning and lenticel damages) was observed in fruits for both conditions (RA and CA). With respect to the results for internal damage, gray flesh was observed in RA-stored fruit after cold storage (13.3%), and the percentage of gray flesh increased to 42.2% at the RTE stage. Vascular browning was also observed at the RTE stage (15.6%). For the fruits submitted to a CA, they did not show these types of disorders.

Regarding pathological problems in this research, we observed mold development at the stem end mainly in CA-stored fruit after 30 days of cold storage, having a 33.3% rate of incidence, while reaching only 6.67% for RA storage. After 50 days, 50% of the fruits in the CA were affected by mold presence. In our research, we observed a slight incidence of stem end rot (<5%) for all fruits analyzed.

#### 2.1.4. Skin Color Development and Skin Color Quality

**Figure 2:**
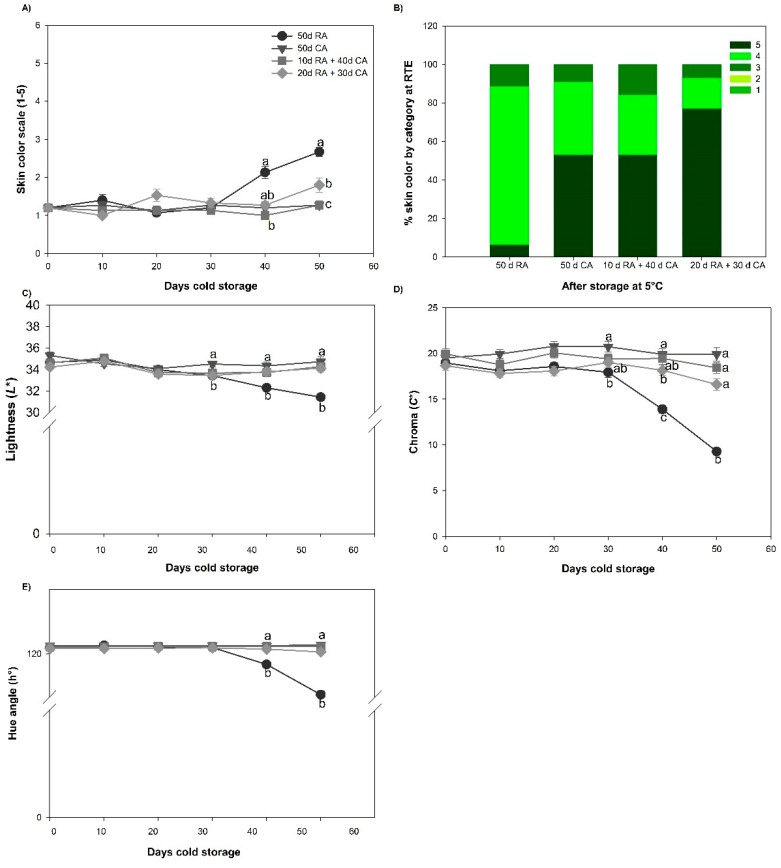
Skin color development and skin color quality of avocado fruit subjected to different treatments. (**A**) Skin color, (**B**) % skin color by category at RTE, (**C**) lightness (L*), (**D**) chroma, (C*) and (**E**) hue angle (h°). Data represent the means of treatments ± SE. Different letters indicate significant differences between treatments for each evaluation time (*p* < 0.05).

The skin of the fruits in all treatment groups did not change during the first 30 days of cold storage. These fruits were classified as level one on the visual color scale (Figure 2A). On the other hand, the color of the RA-stored fruits increased after 40 days of cold storage (level two), and after 50 days changed to level three. CA-stored fruit with a 20-day delay had a mean visual color change of 1.5 after 50 days. For the other treatments, such as the CA (with no delay) and CA with a 10-day delay, fruits did not develop any color change (level one; Figure 2A) after being removed from prolonged storage. However, in the RTE stage, fruits stored in a CA showed greater color development, reaching level five, (>50%) compared to the fruits stored in RA, where only 10% of the fruits showed color development at level five (Figure 2B).

Finally, for color quality, lightness (L*), chroma (C*) and hue angle (h°), there were no significant changes (*p* < 0.05) during cold storage (after 50 days) in the CA-stored fruit, with these treatments attaining values similar to those at harvest. For fruits stored in the RA, L* decreased slightly after 40 days of cold storage and C* changed more rapidly. After cold storage, C* values were half of those at harvest. On the other hand, h° had a slight decrease that was observed after 30 days of cold storage, which coincided with changes in skin color development from level one (green) to level five (purple/black).

### 2.2. Determination of Pigments: Total Chlorophyll, Carotenoid and Anthocyanin Contents

#### 2.2.1. Total Chlorophyll and Carotenoid Contents

**Figure 3:**
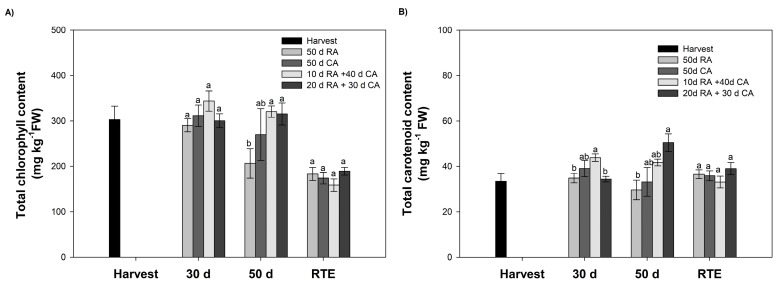
Chlorophyll and carotenoid contents in the skin of avocados subjected to different treatments. (**A**) Total chlorophyll content. (**B**) Total carotenoid content. Bars represent the means of treatments ± SE. Different letters indicate significant differences between treatments for each evaluation time (*p* < 0.05).

In this study, total chlorophyll and carotenoid contents were quantified at harvest, at 30 and 50 days during cold storage and at the RTE stage. The total chlorophyll content decreased as the fruits ripened. At harvest, fruits presented values corresponding to 303.1 mg kg^−1^ of fresh weight (FW) and 158.7 mg kg^−1^ FW at the RTE stage (Figure 3A). Regarding the type of storage (CA or RA), no significant differences in photosynthetic pigments were observed between fruits stored after 30 days (Figure 3A). However, at the end of storage (50 days), fruits in a CA contained higher levels of total chlorophyll than those in the RA, with no significant differences observed between treatments in the CA (Figure 3A). The carotenoid content did not significantly change (*p* < 0.05) during storage (Figure 3B).

#### 2.2.2. Anthocyanin Content

Anthocyanins are the main pigments in the peel of ‘Hass’ avocado [23] and generate a purple/black color, with cyanidin-3-*O*-glucoside [4] standing out. In this research, we identified cyanidin-3-*O*-glucoside as a single anthocyanin in avocado skin tissue (Figure S2). The anthocyanin content was quantified at the same evaluation time (harvest, 30 days and 50 days of storage and the RTE stage) as the chlorophyll content. After 30 days of cold storage, along with the onset of color breakage, a low concentration of cyanidin-3-*O*-glucoside was found in the RA-treated fruit (Table 1). Under CA-stored conditions, no anthocyanins were detected during cold storage. A similar trend was observed in the RA at 50 days of cold storage, but the concentration of the pigment in the fruit was clearly greater. This increase coincided with the observed color changes from level one to level two of the hedonic scale, due to the decrease in chlorophyll and anthocyanin accumulation. At the RTE stage, the anthocyanin content increased approximately five-fold in the CA-stored fruits and CA-stored fruits after 10 days than in RA-stored fruits (Table 1). However, after a delay of 20 days, the CA-treated fruit presented a significantly greater increase (13-fold) in anthocyanin content (Table 1) compared to RA fruit.

### 2.3. Determination of Total Phenolic Compound Content and Antioxidant Capacity

#### 2.3.1. Total Phenolic Content (TPC)

**Figure 4:**
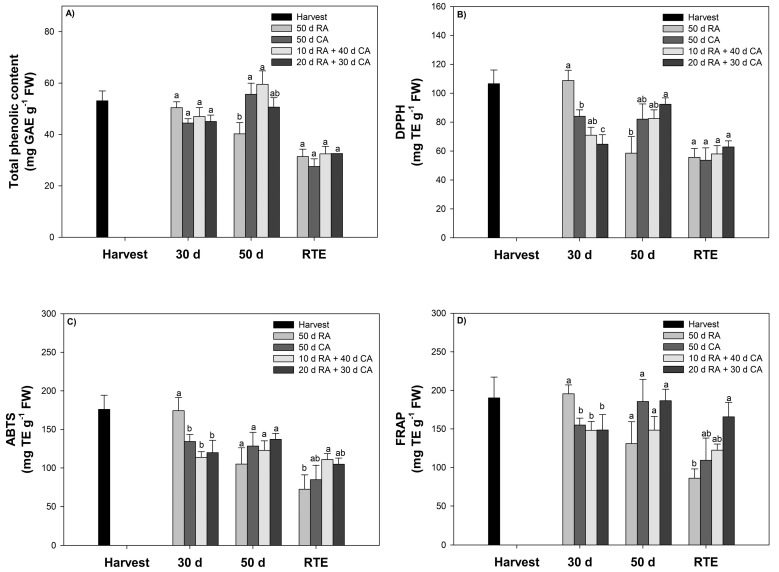
Total phenolic content and antioxidant capacity of avocado skin subjected to different treatments. (**A**) Total phenolic content, (**B**) DPPH, (**C**) ABTS and (**D**) FRAP. Bars represent the means of treatments ± SE. Different letters indicate significant differences between treatments for each evaluation time (*p* < 0.05).

The TPC in the skin was evaluated at harvest, 30 days and 50 days after cold storage (5 °C) and at the RTE stage (Figure 4A). The TPC of the RA-stored fruit ranged from 31.4 to 50.4 mg GAE g^−1^ FW, and for the CA-stored fruit ranged from 27.5 to 59.3 mg GAE g^−1^ FW. After 30 days of cold storage, the content was similar to that at harvest (~48 mg GAE g^−1^ FW). At the end of storage (50 days), the CA-stored fruit contained a greater percentage of TPC than the RA-stored fruit (27%). When the fruits were exposed to a 20-day delay in storage in the CA, the TPC decreased. However, at the RTE stage, there was no significant difference (*p* < 0.05) between fruits stored under a RA or CA (Figure 4A).

#### 2.3.2. Antioxidant Capacity (AC)

AC in avocado skin was evaluated at the same evaluation time as TPC using the following methods: DPPH (radical scavenging), ABTS (cation scavenging, forming a blue–green stable radical cationic chromophore) and FRAP (reduction of ferricyanide ions to ferrocyanide). The results obtained with the three methods showed similar trends. At the harvest stage, higher values of 110 (DPPH assay), 175 (ABTS assay) and 200 mg TE g^−1^ FW (FRAP assay) were detected. Similar values were found for fruit stored under CA conditions. The CA performance of cold-stored fruit showed a slight decrease with time.

## 3. Discussion

The effects of delaying the storage of ‘Hass’ avocados in a CA on the physiological and quality parameters, bioactive compounds and antioxidant capacity of the fruit were evaluated. In the present study, the level of ethylene production was low for all treatments (RA and CA). After 30 days of storage at 5 °C, twice as much ethylene was found in the RA-stored fruits than in the fruits stored under a CA (Figure 1A). The increase in ethylene production in fruits stored under RA conditions could be due to an advanced ripening process, which was consistent with the findings of our previous study [9,24], and occurred because RA technology cannot delay the ripening process; however, CA can delay the onset of climacteric ethylene production [18]. Other cultivars stored under RA or CA, such as ‘Edranol’ and ‘Fuerte’, exhibited similar patterns of ethylene production [9]. With respect to the respiration rate, the level was greater in fruits stored under the CA than in those stored under a RA (Figure 1B). Although a CA is a widely used technology for controlling respiration rates, its efficacy is affected by avocado maturity and cultivar differences, which can lead to CO_2_ and ethylene accumulation if the gas exchange is insufficient [21,22]; as a result, the rates of fruit ripening and senescence are impacted. In addition, Alamar et al. [19] observed that ‘Hass’ avocados stored immediately after harvest in a CA at 5 °C had a greater respiration rate than those stored in RA at 5 °C after 20 days until the end of cold storage (30 days). According to Alamar et al. [19], this increase in the respiration rate during CA storage can be attributed to a stress response of the fruit, which substantially increased CO_2_ levels. This result corresponded with the findings of other studies (e.g., tomato and mango), in which the application of abiotic treatments caused increases in stress-related gene expression and respiration rates [25,26]. For the RA-stored fruits, we observed a slight increase in the respiration rate during storage, peaking after 50 days, which was concomitant with the peak in ethylene production (Figure 1A,B). Previous studies have shown that the respiration rate increases with ripening progression in RA-stored fruit in others cultivars (‘Edranol’ and ‘Fuerte’) [9,24].

In this study, the quality attributes that characterize avocado ripening during postharvest cold storage were fruit mesocarp firmness and skin color. It has been demonstrated that the decrease in mesocarp firmness originates from the activity of enzymes involved in cell wall modification [27,28]. At the harvest stage, mesocarp firmness, which is assessed using nondestructive compression force, generally ranges between 80 and 100 N and gradually decreases during cold storage. Throughout the shelf-life period, the rate of softening increases, resulting in values below 5 N [29,30]. Avocadoes reaching the RTE stage had firmness values between 4 and 14 N [17]. In our study, the mesocarp firmness did not differ between the CA and RA samples after 20 days of storage (Figure 1C). After 30 days of cold storage, the parameter value decreased (near 200 N). Similar effects were reported by Olivares et al. [9,18] and Alamar et al. [19], in which RA-stored fruit softened rapidly for 35 days at 5 °C (3–4 days to reach the RTE stage). Furthermore, Burdon et al. [20], reported that CA-stored fruit ripened more slowly than RA-stored fruit. Similar results were obtained by Hernández et al. [31], who evaluated 12 orchards at early and medium harvests (dry matter respect) and observed that during CA storage, the fruits presented more days of the RTE stage. The ripening of RA-stored fruits was faster than that of CA-stored fruits because during cold air storage (RA), the enzyme system involved in cell wall remodeling continues to function, resulting in a more active enzyme at the beginning of the shelf life. Therefore, when the temperature is increased during the shelf-life process, fruit softening is triggered by this higher level of enzyme activation than when stored in a CA, which results in an inactive and slightly active enzyme system during cold storage [31].

Exocarp browning or lenticel damage was not observed in fruit under either CA or RA conditions during storage (50 days) or after cold storage (shelf-life time). Our results were similar to those reported by Burdon et al. [20], where the incidence of these disorders in RA-stored fruit (62% gray flesh and 95% vascular browning) was significantly greater than that obtained in this study. However, unlike Burdon et al. [20], who showed an incidence of gray flesh (<1%) and vascular browning (39%) in CA-stored fruit, we did not observe these types of disorders. As mentioned above, these differences may result from differences in avocado variety, maturity stag and pre- and postharvest handling [9]. Researchers have observed that ‘Hass’ fruits harvested late in the season, which are characterized by a higher oil content, tend to have a greater incidence of disorders induced by cold storage [32]. Therefore, maturity at harvest and at the ripening stage are critical factors that determine the susceptibility of avocado to the development of physiological disorders [24]. As already mentioned, the most important pathological problem was the development of mold at the peduncular scar. These results contrasted with those reported in other studies, such as those of Burdon et al. [20], in which the main cause was stem end rot. These authors observed a greater incidence in RA-stored fruit (76%) than in CA-stored fruit (46%). In addition, nonsignificant differences were observed between treatments under CA storage (*p* < 0.05). Although different studies have described technologies such as a CA and 1-methylcyclopropene (1-MCP) as useful tools for reducing the incidence of physiological and pathological disorders, critical factors specific to fruit development can influence the efficacy of these tools.

All the avocado fruits were firm and green in color at the time of commercial harvest, which was evaluated on the hedonic color scale as level one, but the color changed as the fruit ripened, decreasing in firmness to 4–14 N and changing the skin color to a dark purple/black color at RTE to level five according to the scale. However, due to the high fruit metabolism of Hass avocados, to preserve quality attributes (e.g., firmness and color), it is necessary to store the fruit in cold storage under regular air conditions (RA) for the local market or in combination with CA storage for export, as this reduces respiration and ethylene production and delays ripening [17]. In this study, CA-fruit and those with a delay of 10 days did not change color during storage, unlike in the other treatments, in which the fruits showed color development, which could be due to the fruits being at a more advanced stage of ripening (Figure 2A). Similar results were reported by Burdon et al. [20], who observed that fruits stored under CA conditions had less skin color development than fruits stored in the RA, but fruits exposed with a 15-day delay in storage in the CA exhibited greater skin color development than fruits stored in other delay periods (1, 5 and 10 days). In contrast to this greater color development in the RA-stored fruit, the CA-stored fruit showed greater color development at the RTE stage (Figure 2B). Burdon et al. [20] found that when the fruit was subjected to a longer delay time to storage in a CA (15 days), better color development occurred during this stage. In a previous study characterizing the ethylene response in Hass avocados, we demonstrated that after 20 days of storage, a greater number of enzymes and genes related to metabolic processes, including cell wall biogenesis, intracellular signal transduction, phenylpropanoid metabolic processes and the response to ethylene, were activated [18]. Additionally, cold storage induced the expression levels of all genes involved in ethylene synthesis and perception, which were much greater after 21 days at 5 °C, concomitant with higher levels of ethylene and increased softening of the fruit [18]. Therefore, a delay in the CA close to 20 days would not be able to slow the already triggered ripening process.

As already mentioned, as the ‘Hass’ avocado reaches consumption maturity, the color of its exocarp changes from green to black or dark purple. Chlorophylls are responsible for the color transition to green shades, which decrease as the fruit ripens, and anthocyanins are responsible for the color transition to purple/black shades, which accumulate during ripening [4,13].

In line with this, our results indicated a decrease in the chlorophyll content as the fruit ripened (Figure 3A). These results resembled those reported by Cox et al. [4], who observed decreases in chlorophyll as fruits ripened at different temperatures (15, 20 and 25 °C). However, these fruits were not placed under prolonged storage, as in our experimental setup. On the other hand, Arancibia-Guerra et al. [1] showed no decreasing trends in total chlorophyll or total carotenoid concentrations among the different sampling times (harvest, 40 days and RTE stage) or storage types (CA and RA), revealing significant differences only for fruits with higher dry matter (>30%) contents, which contained higher chlorophyll and carotenoid contents. Despite the discrepancy between the results, both studies reported chlorophyll contents similar to our results. Regarding the anthocyanin content, we agreed with other authors and identified one anthocyanin, cyanidin-3-*O*-glucoside [1,4,13] (Figure S2). The accumulation of cyanidin-3-*O*-glucoside coincided with the observed color changes from green (level 1) to purple/black (level 4/5). Arancibia-Guerra et al. [1] reported a positive correlation between the anthocyanin content and color accumulation in the skin. In this context, our results were similar to those obtained by this author, who reported that fruits stored for 40 days under RA and CA conditions did not contain anthocyanins. Similarly, the content increased in the RTE stage, but mostly without significant differences (except for fruit from one orchard) in the black-colored phenotype between the CA and RA storage conditions. This finding differs from our results, as we observed a significantly greater concentration of the pigment at the RTE stage than in the other treatments (Table 1). This behavior may have resulted from the time that the fruit in this treatment was exposed to RA, as all the biochemical processes that lead to consumption maturity, such as color change, were triggered; this activity coincided with changes in the development of skin color and a decrease in color quality parameters. In addition, the behavior observed for CA after a 20-day delay may also have been influenced by the DM content (>30%). Similar results were presented by Arancibia-Guerra et al. [1], who reported that the anthocyanin content increases directly with the DM content. Additionally, for other species, such as apple, in which the time of harvest positively regulates anthocyanin accumulation in the skin [33], the anthocyanin content is influenced by and/or controlled by hormones.

In our study, the TPC of the fruit was similar to that described by Lyu et al. [7], who obtained a range of 29.2–77.9 mg GAE g^−1^, and Rodriguez-Carpera et al. [34], who reported a concentration of 78.41 mg GAE g^−1^ (methanol solvent); in contrast, our values were greater [8,35,36]. According to these publications, the range of TPC found in skin is wide, from 1.81 to 227 mg GAE g^−1^ (Figure 4A). The TPC of avocado skin reported in this and other studies was several times greater than that of other tropical fruits [7,8,34]. At 50 days, CA-stored fruits had a greater TPC than RA-stored fruits. Similar results were reported by Fuentealba et al. [37], as CA-stored fruits displayed a greater TPC, with a difference observed between harvest seasons; this difference was greater for fruits that presented a greater DM content (>26–30%). On the other hand, the effect of the CA at different O_2_ concentrations on the total phenolic content was also evaluated in other species, such as jackfruit; it was observed that a CA with high levels of CO_2_ and low O_2_ better maintained phenolic compounds than other conditions [38]. The TPC of avocado skin can change in response to different factors, such as variety or cultivar, cultural management, climatic conditions, postharvest technologies, maturity stage and methodology used for the analysis [7,36,39]. In addition, the phenolic compounds and other polar compounds in avocado skin are lower in overripe avocado than in optimally ripened avocado due to the presence of phenylalanine ammonium-lyase (PAL), which can increase its activity with fruit ripening and increase its concentration of phenolic compounds [40]. Several factors, including intrinsic (such as genus, species and cultivar) and extrinsic (including agronomic aspects, environmental conditions, handling and storage) factors, among others, affect the amount and composition of phenolic compounds present in foods and plants [41]. Our results for antioxidant capacity and TPC were high. In CA fruit, the antioxidant capacity decreased slightly during storage (Figure 4B–D). Similar results were reported by Fuentealba et al. [37], as fruits with a lower DM (23–26%), i.e., from an early harvest, showed a greater decrease in the CA after 40 days. Lyu et al. [7] reported that the maturity stage modified the antioxidant capacity and that immature fruits had the lowest values of 57.82 (DPPH), 39.05 (ABTS) and 1.00 (FRAP) mg ascorbic acid equivalent g^−1^ of the sample (fresh weight). These changes in antioxidant capacity could be explained by the different degrees to which polyphenolic compounds were polymerized, which were affected by cold storage conditions [42]. This difference could be related to a series of chemical and enzymatic modifications that occur during the ripening phase of the fruit, such as the hydrolysis of glycosides by glycosidases, oxidation of phenols by phenol oxidases and polymerization of free phenols [37]. Moreover, compared with those of other authors, the range of values could have been affected by many factors, such as the solvent, temperature, chemical structure of phenolic compounds and pH, which could have affected the antioxidant mechanism and the accuracy of the activity estimation [7]. Finally, the skin is the main barrier presented by the fruit due to exogenous agents, such as sunlight, which triggers a massive generation of phenolic compounds to prevent oxidative damage [43,44].

## 4. Materials and Methods

### 4.1. Materials

Four hundred avocados were collected from a commercial orchard located at Panquehue (Valparaiso Region, Chile). ‘Hass’ avocado fruits were harvested with a dry matter content higher than 30%. The harvest conditions were determined by sampling 20 fruits for dry matter results, as commercially practiced. Fruits were immediately transported to Postharvest Laboratory facilities at the Instituto de Investigaciones Agropecuarias (INIA, Santiago, Chile).

### 4.2. Dry Matter (DM)

The DM content (harvest stage) was determined by drying the mesocarp samples in an oven at 103 °C until a constant weight was attained. The results were expressed as a percentage (g of DM per 100 g of mesocarp) [9,45].

### 4.3. Postharvest Treatments

A total of three hundred and eighty fruits were selected for all treatments, and another 20 fruits were evaluated (harvest time). The fruits were divided into 4 treatment groups as follows: (i) 50 days of storage in regular air (RA, 21 kPa O_2_ and 0.04 kPa CO_2_), [37] (ii) 50 days of storage in a CA of 4 kPa O_2_ and 6 kPa CO_2_ using a static automatic gas CA unit (Kronenberger System Technik, Friedrichshafen, Germany), (iii) 10 days of storage in a RA plus 40 days in a CA and (iv) 20 days of storage in a RA plus 30 days in a CA. For each treatment, 90 fruits were packaged in three ventilated plastic boxes (n = 30 avocados) and stored at 5 °C for 50 days. The relative humidity (RH) values for the RA were 80–85%, and those for the CA were >90%. Evaluations were performed during cold storage every 10 days (10 days, 20 days, 30 days, 40 days and 50 days); after cold storage, the fruit samples were maintained at shelf life (20 °C and 45% RH) until they reached the ready-to-eat (RTE) stage (mesocarp firmness 4–14 N) [31]. For each evaluation time, exocarp fruit samples were frozen in liquid nitrogen and stored at −80 °C.

### 4.4. Physiological and Quality Parameters

#### 4.4.1. Respiration and Ethylene Production Rates

The respiration and ethylene production rates were measured for 15 individual fruits per treatment (5 fruits per replicate; afterwards, these fruits were evaluated for the rest of the analysis). The respiration rate and ethylene production measurements, the fruits were placed in a plastic container (1.6 L) and sealed for 2 h at 20 °C to prevent the excessive accumulation of CO_2_. The respiration rate was measured by injecting 1 mL of gas from the headspace into a gas analyzer (PBI-Dansensor Checkmate 9900, Ringsted, Denmark), and the values were expressed as mL CO_2_ kg^−1^ h^−1^ [9]. To measure ethylene production, 1 mL of gas was removed from the headspace and injected into a gas chromatograph (Shimadzu GC 8A, Tokyo, Japan), as described by Olivares et al. [9]. The values were expressed as μL C_2_H_4_ kg^−1^ h^−1^.

#### 4.4.2. Mesocarp Firmness

Mesocarp firmness was measured with a penetrometer (Effegi, FT327, FT011, Alfonsine, Italy) equipped with a 4 mm (for firm fruit) or 8 mm (for the RTE stage) plunger tip. The measurements were conducted on two sides of individual fruits (n = 15 for each treatment) from the equatorial zone at room temperature. Firmness measurements were performed at harvest, every 10 days of storage and at the end of shelf life. The skin was removed before the firmness evaluation, and the values were expressed in Newton (N).

### 4.5. Physiological and Pathological Damage

The physiological damage included external (exocarp browning and lenticel damage) and internal (gray pulp and vascular browning) disorders. A hedonic scale was used according to García-Rojas et al. [24], ranging from 1 to 5 (1: no occurrence; 2: slight; 3: moderate; 4: moderately severe; and 5: severe). The pathological damage evaluated included mold and stem end rot. These evaluations were performed at harvest, every 10 days of storage and at the end of shelf life (n = 15 avocados per treatment). The results were expressed as the incidence (%) of damage.

### 4.6. Skin Color Development and Skin Color Quality

Skin color development was assessed visually using a hedonic scale (from 1 to 5) according to Rivera et al. [2], where:

1 = 100% of the skin surface was green;
2 = 20% of the skin surface was colored purple/black (violet) on the green;
3 = 60% of the skin surface was colored purple/black (violet) on the green;
4 = 100% of the skin surface was purple (violet);
5 = 100% of the exocarp surface was black.

The skin color quality was assessed using a reflectance colorimeter (CR-300, Minolta, Japan) with the CIELAB color system. Two equidistant color measurements were carried out around the equatorial zone of each fruit (n = 30 for each treatment), and the L*, a*, b*, C* and h° units of the color space were used. L* is the lightness (0 = black; 100 = white); C* is the chroma, a measure of the intensity/purity of color (0 = achromatic); a* and b* are the chromatic coordinates representing the red–green and yellow–blue axes, respectively; and h° is the hue angle on the color wheel (0° = red; 90° = yellow; 180° = green; 270° = blue) [46].

### 4.7. Preparation of Exocarp Fruit Samples

Based on the results of the physiological and quality parameters, to determine the bioactive compounds and antioxidant capacity, the following evaluation times were selected: at harvest, 30 days and 50 days and the RTE stage. Exocarp fruit samples were frozen in liquid nitrogen and then lyophilized at −55 °C under vacuum (Liotop L101, Liobras, Brazil) for 72 h. Exocarp fruit samples were weighed before and after the lyophilization process to calculate the moisture content and transformed from dry weight (DW) to FW. The lyophilized samples were packed in vacuum and stored at room temperature until analyzed.

### 4.8. Determination of Pigments: Chlorophyll, Carotenoid and Anthocyanin Contents

#### 4.8.1. Total Chlorophyll and Carotenoid Contents

Chlorophyll and carotenoid extraction was performed using a 50 mg lyophilized exocarp sample, methanol as the extracting agent and absorbance at 665, 652 and 470 nm, according to Miazek and Ledakowicz [47], with some modifications. The final content was calculated using the following equations proposed by Lichtenthaler [48]:

(1) $$Total\;Chlorophylls\left(\mu g\;{mL}^{-1}\right)=\left(1.44\times A_{665}\right)+\left(24.93\times A_{652}\right)$$

(2) $$Chlorophyll\;a\left(\mu g\;{mL}^{-1}\right)=\left(16.72\times A_{665}\right)-\left(9.16\times A_{652}\right)$$

(3) $$Chlorophyll\;b\left(\mu g\;{mL}^{-1}\right)=\left(34.09\times A_{652}\right)-\left(15.28\times A_{665}\right)$$

(4) $$Total\;Carotenoids\left(\mu g\;{mL}^{-1}\right)=\left(\left(1000\times 470\right)-\left(1.63\times C_{a}\right)-\left(104.9\times C_{b}\right)\right)\times 221^{-1}$$

The results were corrected using the concentration of the samples (g mL^−1^) and the final concentration of pigments was calculated as mg kg^−1^ fresh weight (FW) sample.

#### 4.8.2. Anthocyanin Content

Anthocyanin extraction was performed using 100 mg of previously ground lyophilized exocarp, and 5 mL of acidified methanol (10% *v*/*v* acetic acid in methanol and 0.2% trifluoroacetic acid (TFA)) was added and vortexed for 1 min. This mixture was then centrifuged at 1000 RPM at 20 °C for 10 min, after which the supernatant was filtered through a 0.45 mm syringe filter and stored at −80 °C until analysis.

Anthocyanin quantification was performed with an HPLC-DAD chromatographic system (Jasco interface LC-NetII/ADC 7059-J012A, detector MD-4010 quaternary pump PU-4180-LPG, column oven CO-4060, autosampler AS-4050 with cooling system TC-4000-1, Jasco, Tokyo, Japan), C18 column PerkinElmer LC (250 × 4.6 mm, 5 µm), according to Vergara et al. [49], with modifications. The samples (20 µL) were then injected. The mobile phases A (0.2% v/v TFA in water), B (0.2% v/v TFA in acetonitrile) and C (0.2% v/v TFA in methanol) were used under the following conditions: initial, 7% B and C, followed by a linear change to 13% B and C for 40 min. The column oven temperature was 40 °C. The quantification was carried out using a calibration curve of cyanidin-3-*O*-glucoside as a standard. The results were expressed as mg cyanidin-3-*O*-glucoside kg^−1^ FW.

### 4.9. Determination of Total Phenolic Content and Antioxidant Capacity

To determine the total phenolic content (TPC) and antioxidant capacity (AC), a method described by Fuentealba et al. [37] was used with some modifications. Briefly, 50 mg of lyophilized exocarp tissue was homogenized with 2 mL of methanol and mixed in a shaker (Shaking incubatorLabTech, Batam, Indonesia) at 150 RPM and a 20 mm orbital motion shaker orbit for 1 h. Then, the mixture was centrifuged at 13,500 RPM and 4 °C for 20 min (Labnet International, Inc., Edison, NJ, USA). Two hundred microliters of the supernatant was evaporated with a speed vacuum centrifuge and the remaining pellet was resuspended in 400 µL of methanol and stored (−20 °C) for a subsequent analysis of TPC and AC. The total phenolic content and antioxidant capacity (DPPH, FRAP, ABTS) were determined according to the methods of Lyu et al. [7]. Each sample was analyzed in triplicate and absorption data were measured with an Epoch Bioteck microplate spectrophotometer (Bioteck, Waltham, MA, USA).

#### 4.9.1. Total Phenolic Content Analysis

TPC was quantified by mixing 240 μL of water (HPLC grade), 20 μL of Folin–Ciocalteu reagent (1 N), 20 μL of 5% (*w*/*v*) sodium carbonate and 20 μL of the extract in microplates. The samples were incubated for 2 h in darkness at room temperature, after which the absorbance was measured at 765 nm. The TPC was expressed as the mg gallic acid equivalent (GAE) g^−1^ of the FW sample.

#### 4.9.2. Antioxidant Method

##### DPPH (2,2′-Diphenyl-1-picrylhydrazyl) Assay

A mixture of 15 µL of the extract and 285 µL of DPPH (0.12 mM) was placed in a microplate. The absorbance was measured at 517 nm after 30 min of incubation in the dark. The Trolox calibration curve was prepared with concentrations ranging from 0 to 250 ppm. The DPPH value was expressed as the mg Trolox equivalent (TE) g^−1^ of the FW sample.

##### ABTS Assay

The ABTS solution was prepared by mixing 7 mM ABTS with 2.45 mM potassium persulfate in equal amounts. The solution was incubated at room temperature for 16 h, after which the absorbance was adjusted to 0.7 with methanol. For the reaction, 15 µL of the sample and 285 µL of the ABTS solution were added to the microplate and incubated for 7 min in the dark, after which the absorbance was measured at 734 nm. A Trolox calibration curve was prepared with concentrations ranging from 0 to 280 ppm. The ABTS values are expressed as the mg TE g^−1^ FW sample.

##### Ferric Reducing Antioxidant Power (FRAP) Assay

The FRAP reagent was prepared by mixing 25 mL of acetate buffer (pH 3.6) with 2.5 mL of 2,4,6-tripyridyl-S-triazine (TPTZ) and 2.5 mL of FeCl_3_. For the reaction, 15 µL of the sample and 285 µL of FRAP solution were added to the microplate. The absorbance was measured at 593 nm after 30 min of incubation in the dark. A Trolox calibration curve was prepared with concentrations ranging from 0 to 280 ppm. FRAP values were expressed as the mg TE g^−1^ FW sample.

### 4.10. Experimental Design and Statistical Analysis

The experiments were performed with a completely randomized design. Prior to analyzing the percentages, the data were arcsine transformed, and non-transformed values were used for presentation in the figures. The data were subjected to a statistical analysis of variance (ANOVA), and the means were separated through a least significant difference (LSD) test at 5% significance using the statistical software InfoStat (version 2015, Universidad Nacional de Córdoba, Córdoba, Argentina).

## 5. Conclusions

This study showed that a 20-day delay between harvest and CA storage for transit in a container had a detrimental effect on increasing the fruit softening rate during cold storage, reducing the effectiveness of the CA as a postharvest technology for extending storage life. This effect was also observed in the reduction in days to reach the RTE stage at 20 °C by enhancing the skin color development compared to CA stablished immediately after harvest. This increase in color development was concomitant with a higher concentration of cyanidin-3-*O*-glucoside, the most important anthocyanin for ‘Hass’ avocado, an increase in total phenolic content and a reduction in total chlorophylls. Thus, despite the advantage of the CA in extending ‘Hass’ avocado shelf life, efforts must be performed for optimizing its benefits, for reducing softening and color development rates, throughout the immediate CA establishment after avocado harvest, as shown in this study.

## Figures and Tables

**Table 1:** Anthocyanin content (mg cyanidin-3-*O*-glucoside g^−1^ FW) at harvest, after postharvest treatment and at the corresponding RTE stage.

Harvest	ND		
Treatment	30 d at 5 °C	50 d at 5 °C	RTE
RA	0.01 ± 0.0	0.49 ± 0.01 ^a^	0.44 ± 0.03 ^c^
CA	ND	ND	2.22 ± 1.13 ^b^
10 d RA + 40 d CA	ND	ND	2.67 ± 0.56 ^b^
20 d RA + 30 d CA	ND	0.03 ± 0.0 ^b^	5.92 ± 0.33 ^a^

ND: not detected; RA: regular air; CA: controlled atmosphere; RTE: ready-to-eat. Different letters in the same column indicate significant differences between treatments for each evaluation time (*p* < 0.05).

## Data Availability

Data are contained within the article.

## Supplementary Materials

The following supporting information can be downloaded at: https://www.mdpi.com/article/10.3390/plants13111455/s1, Figure S1. Pigments at 50 days of cold storage. (A) Total chlorophyll content. (B) Anthocyanin content. T1: 50 d RA; T2: 50 d CA; T3: 10 d RA + 40 d CA; T4: 20 d RA + 30 d CA; Figure S2: Representative HPLC-DAD chromatogram of cyanidin-3-*O*-glucoside in skin avocado.
