# Supplementary material for: Effects of Delaying the Storage of ‘Hass’ Avocados under a Controlled Atmosphere on Skin Color, Bioactive Compounds and Antioxidant Capacity
Source: Plants (Basel). 2024 May 24;13(11):1455. doi: 10.3390/plants13111455 (PMC11174840; doi:10.3390/plants13111455)
Supplement: Supplementary file 1 [file plants-13-01455-s001.zip › plants-2940945-supplementary.pdf]

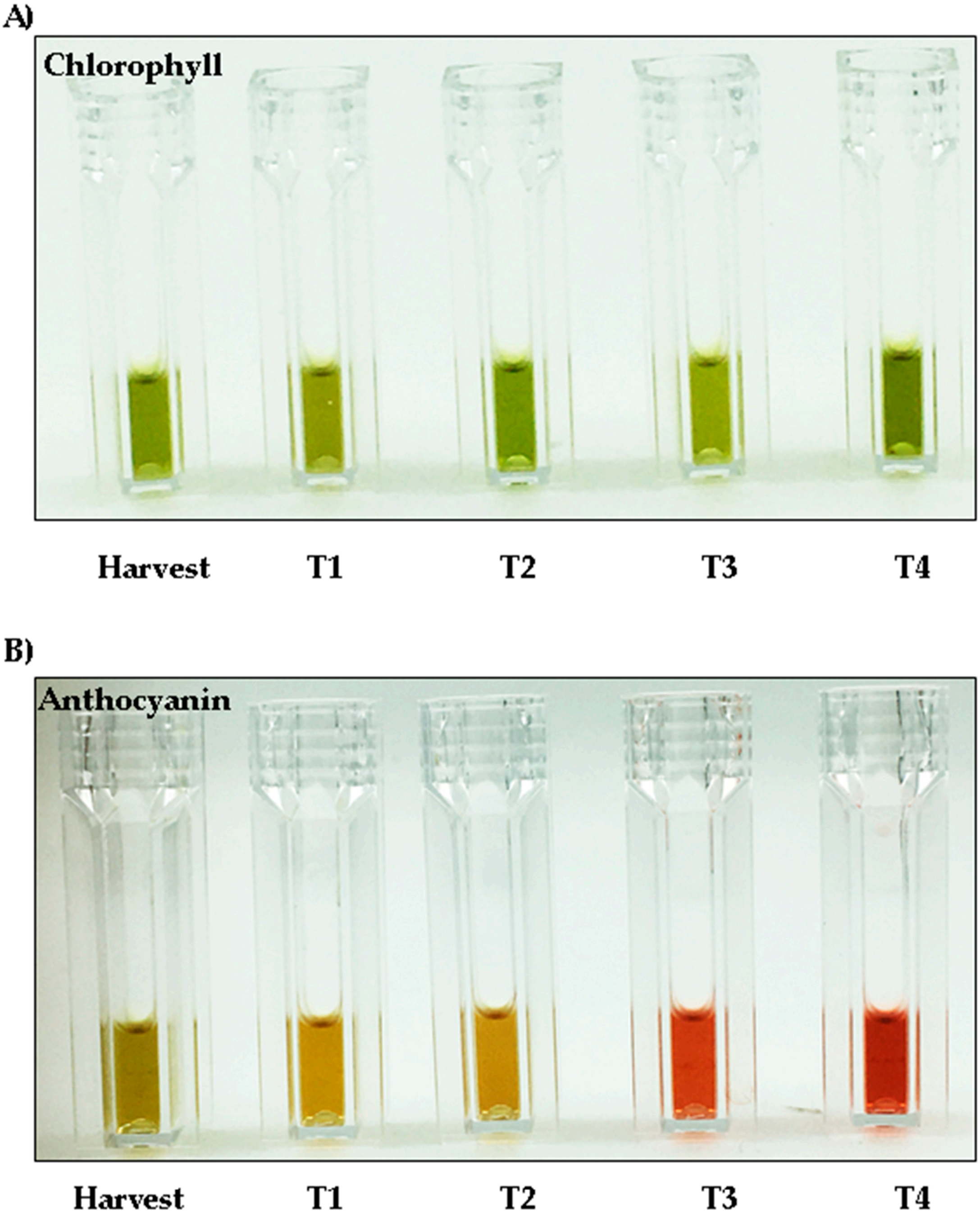

Figure S1. Pigments at 50 days of cold storage. A) Total chlorophyll content. B) Anthocyanin content. T1: 50 d RA; T2: 50 d CA; T3: 10 d RA + 40 d CA; T4: 20 d RA + 30 d CA.

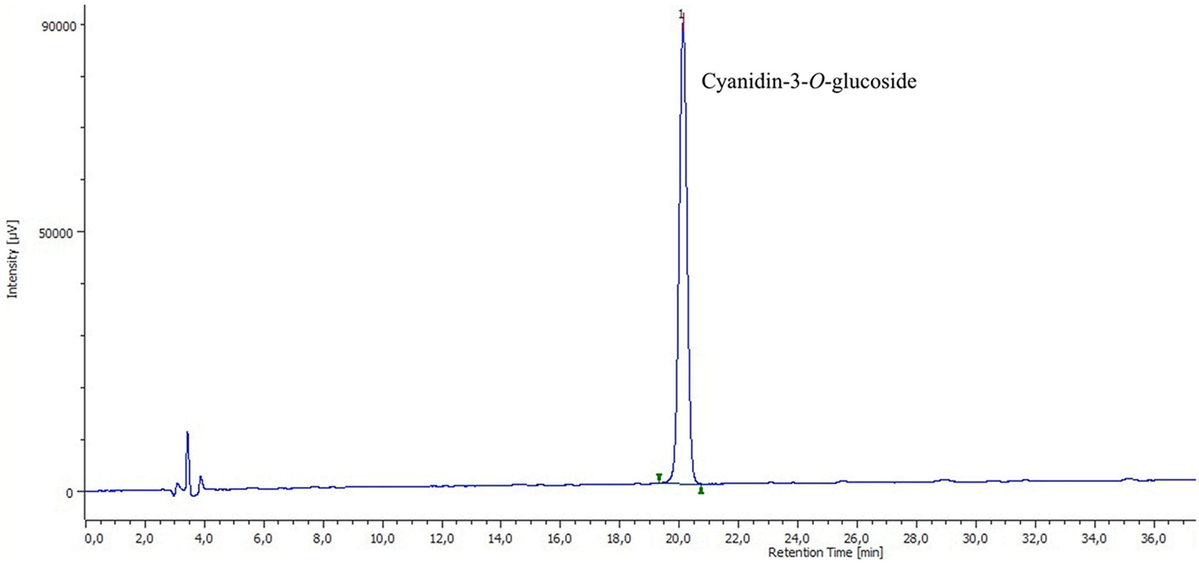

Figure S2. Representative HPLC-DAD chromatogram of cyanidin-3-*O*-glucoside in skin avocado.
